# The hidden curriculum of endurance: how students’ psychosocial stress becomes normalised in arts higher education

**DOI:** 10.1080/17482631.2026.2727777

**Published:** 2026-09-17

**Authors:** Barbora Procházková, Kateřina Zábrodská

**Affiliations:** a Faculty of Social Sciences, Institute of Sociological Studies, Charles University, Prague, Czech Republic; b Institute of Psychology, Czech Academy of Sciences; Department of Psychology, Faculty of Arts, Charles University, Prague, Czech Republic

**Keywords:** Arts higher education, psychosocial stressors, student well-being, student mental health, hidden curriculum, professional socialisation

## Abstract

**Purpose:**

The deterioration of university students’ mental health has become a global public health concern. However, students’ well-being in arts higher education remains underexamined, despite evidence that arts students may be especially vulnerable to psychosocial distress. This study explored how students in artistic disciplines experienced psychosocial stressors related to their studies in Czech arts higher education.

**Methods:**

Thirtysemi-structured interviews with students enrolled in visual, audiovisual, and performing arts programmes were analysed using reflexive thematic analysis.

**Results:**

Three categories of major psychosocial stressors were identified: institutional, organisational, and interpersonal. Institutional stressors included norms of total commitment, meritocratic constructions of success, and pressure to accumulate social capital. Organisational stressors involved intensive time demands, ambiguous and subjective evaluation practices, and emotionally demanding Final Critiques. Interpersonal stressors involved blurred student−teacher boundaries, intergenerational tensions, and competitive peer dynamics. Across these stressors, a hidden curriculum of endurance emerged through which exhaustion, uncertainty, and self-sacrifice became normalised as part of becoming an artist.

**Conclusions:**

The findings show that psychosocial stressors in arts higher education are socially and institutionally produced and embedded in the everyday conditions of artistic training. Effective responses should address the organisational cultures, pedagogical practices, and relational dynamics through which psychosocial stress is produced and normalised.

## Introduction

Growing concern over university students’ mental health has become increasingly visible across national and institutional contexts, raising urgent questions about how higher education environments impact students’ experiences during a life stage marked by intense academic, social, and personal transitions (Emmerton et al., 2024; Mahieu & Erbach, 2023; Sivertsen et al., 2025). Across the European Union, approximately 40% of university students report mental health difficulties (Cuppen et al., 2024). Global evidence similarly indicates a high burden of distress among university students: a recent umbrella review synthesising 62 meta-analyses reported prevalence estimates of 40.2% for anxiety symptoms, 36.6% for stress, and 35.4% for depressive symptoms (Paiva et al., 2025). Seeking to identify the sources of students’ distress, previous research has identified a wide range of both academic and non-academic stressors, such as performance pressure, demanding adaptation, financial strain, relationship difficulties, and job insecurity (see, e.g., Barbayannis et al., 2022). Studies have also examined students’ coping strategies, distinguishing adaptive from maladaptive forms of coping (Freire et al., 2020).

Although this body of work has generated important insights, much of it has relied on quantitative designs focused on identifying stressors and individual coping responses. Comparatively less is known about how students subjectively experience and interpret these demands and how their stress is shaped by the institutional and organisational environments in which they study. Recent scholarship has therefore called for qualitative approaches capable of examining these contextual and meaning-making processes (Majerová & Sokolová, 2025; Martínez-García et al., 2024). Responding to this gap, this study aims to expand research on psychosocial stressors among students by shifting attention from individual students’ vulnerability to the institutional production of psychosocial stress in the specific sector of arts higher education.

We focus specifically on arts higher education, as emerging research suggests that students in artistic disciplines experience higher levels of mental distress than students in many other fields (Lee et al., 2024). Drawing on semi-structured interviews with students in visual, audiovisual, and performing arts programmes, we examine how students experience, interpret, and navigate stress within the everyday institutional, organisational, and relational contexts of their studies. Complementing existing research on student mental health and academic stress, which has often approached these issues primarily from psychological perspectives, this study adopts a sociological perspective informed by the stress process model (SPM) (Pearlin & Bierman, 2012; Pearlin et al., 1981), through which student distress is understood as socially patterned and institutionally mediated. Additionally, we draw on critical accounts of meritocracy (Littler, 2017) and on Bourdieu’s concept of social capital (1993; Bourdieu, 1986) to examine how psychosocial stressors are affected by unequal access to opportunities and legitimacy in arts higher education. The study aims to contribute to critical qualitative research on student mental health and well-being by exploring how students’ psychosocial stress is embedded in the everyday conditions of arts higher education. While the study is grounded in the specific national context of the Czech Republic, this Central European context provides an analytically useful case of a small, selective, and closely interconnected arts higher education field whose features may uncover broader patterns of psychosocial stress and professional socialisation.

## Psychosocial stressors in arts higher education

Arts higher education constitutes a distinctive pedagogical and psychosocial context in which academic demands are closely intertwined with creative performance, intense personal investment, and forms of evaluation that may be experienced as emotionally charged and ambiguous (Blevins et al., 2022; Greason et al., 2015). In some disciplines, evaluation involves embodied forms of practice in which physical performance and bodily appearance become salient, increasing students’ vulnerability (Bernhard, 2007; Fostervold Mathisen et al., 2022). These forms of assessment affect students’ academic progress as well as their identities and sense of belonging within the field (Motley, 2017; Shreeve et al., 2010). Moreover, in many European contexts, including Czechia, visual arts education remains structured around studio-based settings characterised by close proximity between students and their teachers and peers, collective critique practices, and ongoing reflection on creative work (Cleary Rodrigues, 2025; Nyboer et al., 2025). These arrangements may heighten students’ dependence on interpersonal relations and increase their exposure to institutional power asymmetries. In performance-based arts disciplines in particular, students are often guided throughout their studies by a single teacher, frequently through one-to-one instruction, an arrangement that can also entail physical isolation (Burwell et al., 2019; Gaunt, 2010). Within these environments, peer relations and informal communities of practice can become materially consequential, as they profoundly influence students’ visibility and access to professional opportunities (Goodwin & Vincent, 2024; Martin et al., 2023). Public presentations and evaluations of students’ work further intensify this dynamic by intertwining assessment, professional socialisation, and the formation of students’ identities as future artists (Blair, 2007; Hastings, 2017; Kornetsky, 2017). Overall, these studies jointly indicate that arts students may be exposed to a distinctive combination of academic, emotional, and professional pressures that cumulatively increase their vulnerability to distress.

These pedagogical and professional characteristics may help explain why students in arts and creative disciplines appear particularly vulnerable to mental health difficulties (Alessandri et al., 2020; Perkins et al., 2017). Arts students have been found to report greater stress, devote more time to academic work, and experience higher levels of anxiety and depression than students in some non-art disciplines (Greason et al., 2015; Lee et al., 2024; Vaag et al., 2021). For example, Vaag et al. (2021) found that Norwegian students in arts and music programmes reported significantly higher levels of mental health difficulties than students in non-art fields, including elevated symptoms of anxiety and depression, a higher prevalence of self-reported mental disorders, and more frequent use of psychotherapy. Similar concerns have been reported among performing arts students (Curtis, 2019). Such heightened vulnerability has been linked to performance pressure, excessive workloads, competitive environments, and the close identification of artistic work with the self (Araújo et al., 2017; Perkins et al., 2017).

## Theoretical framework

This study draws on the stress process model (SPM) (Pearlin & Bierman, 2012; Pearlin et al., 1981), which conceptualises stress as a socially structured process influenced by patterned exposure to stressors and unequal access to coping resources. Within this framework, stressors are defined as experiential or perceived circumstances arising from social environments that become consequential when they are appraised as threatening, burdensome, or as exceeding available coping resources (Pearlin & Bierman, 2012; Pearlin, 1989). In arts higher education, this perspective is particularly useful because it shifts attention away from students’ individual attributes and towards the cultural, organisational, or relational conditions through which distress may be produced. It thus allows institutional norms, organisational arrangements, and pedagogical relationships to be analysed as mechanisms of stress rather than merely as the external context for individual coping. The continued relevance of the SPM in contemporary mental health research, including studies of work-related stress and social inequalities, further supports its analytical usefulness for examining socially patterned experiences of distress in institutional contexts (see, e.g., Fan & Ryu, 2023; Lai et al., 2026; Van Gundy & Burke, 2025).

However, analysing psychosocial stressors in arts higher education requires attention to both exposure to stressors and the normative and symbolic conditions through which these stressors acquire meaning and legitimacy. For this reason, we integrate the SPM with sociological theories that illuminate how artistic education is organised through unequal relations of recognition, value, and belonging. Drawing on Pierre Bourdieu (1986, 1993) arts higher education can be understood as a social field in which access to recognition and future opportunities depends not only on individual performance but also on the unequal distribution of economic, cultural, and social capital. This perspective is particularly relevant in educational contexts in which success is framed formally in terms of individual talent, effort, and artistic commitment but is simultaneously mediated by informal norms, professional visibility, and networked forms of access to the art world. Critical accounts of meritocracy (Littler, 2017; Liu, 2011) further clarify how such arrangements are legitimised. Rather than functioning as a neutral principle of fair reward, meritocracy can operate as a cultural and moral framework that attributes success and failure primarily to individual qualities, thereby obscuring the structural and relational conditions through which opportunities are distributed (Brighouse, 2022; Littler, 2017). These perspectives allow us to conceptualise stress in arts higher education as a socially patterned experience shaped by institutionalised expectations, unequal access to recognition, and the reproduction of structural inequalities.

## National context

The Czech context adds further specificity, as arts higher education is organised within a small, selective, and predominantly public institutional field (Skála & Chylíková, 2022). Arts higher education in Czechia comprises four specialised public higher education institutions involving seven faculties, alongside arts and design programmes offered within comprehensive public universities, as well as two private arts-oriented higher education institutions. Admission is typically based on talent and portfolio assessment and is highly selective: in 2025, admission rates at several major specialised arts faculties and institutions ranged from 10% to 23% (VysokeSkoly.com, 2025).^1^ At the same time, the predominantly public character of the sector means that arts higher education institutions depend heavily on state funding. Against the background of comparatively low education spending in Czechia^2^ (Organisation for Economic Co-operation & Development, 2024), this dependence constrains institutional capacity, including staffing levels and the number of students that institutions are able to admit. High selectivity limited institutional capacity, and the small number of institutions thus contribute to a closely interconnected field in which institutional reputation, visibility, and professional relationships become particularly salient to students’ study experiences and professional formation.

Public attention to students’ mental health in Czech arts higher education has recently increased following student-led mobilisation that brought issues of abuse of power, boundary violations, excessive workload, and lack of psychosocial safety into public debate. These initiatives created space for students to voice their experiences of inappropriate conduct, misuse of institutional power, excessive demands, and mental health concerns. A prominent example was *You Don’t! Have to Endure This!*, which began with an anonymous survey and culminated in a public reading at the Theatre Faculty of the Academy of Performing Arts in Prague in 2021 (Nemusíš to vydržet, 2021). Such initiatives helped transform experiences previously treated as individual problems into matters of public and institutional concern, prompting universities to address study conditions and students’ psychosocial safety. This mobilisation contributed to the wider visibility of mental health and psychosocial safety in arts education as urgent social problems and prompted numerous institutional responses, including the introduction or strengthening of ombudsperson roles, expanded psychological counselling and consultation services, and more formalised procedures for addressing complaints and safeguarding students (Školská Ombuds Platforma, 2026; Smužová, 2022).

## Methodology

### Research aims and question

This study investigated how institutional arrangements and cultural norms within arts higher education become sources of psychosocial stress in students’ everyday lives. It addressed the following research question: *How do students in artistic disciplines experience psychosocial stressors emerging from the institutional environment of arts higher education*?

### Research design and data collection

The study adopted a qualitative design based on semi-structured interviews conducted within a broader research project on psychosocial resilience in higher education (Machovcová et al., 2026). Semi-structured interviews were chosen because they combine comparability across interviews with sufficient flexibility for participants to articulate their subjective experiences in depth (Silverman, 2013). The interviews were conducted by the first author between June 2023 and April 2025. Most took place in person in Prague; eight were conducted online via Google Meet when participants were residing elsewhere in Czechia or abroad. The interview guide focused on participants’ experiences of arts higher education and covered three major topics: the perceived quality of the learning environment (e.g., “How do you perceive the quality of your current studies?”; “In what ways do your studies meet or not meet your expectations?”), the social climate and relationships (e.g., “How would you describe relationships within your department?”; “How do you perceive the social climate in your department?”), and students’ well-being and mental health (e.g., “How would you describe your well-being in relation to your studies?”; “What kinds of stressors, if any, do you experience in relation to your studies?”). The interviews followed a flexible format based on open-ended questions and follow-up prompts and lasted approximately 70 minutes on average.

The interviews were carried out, transcribed, and initially coded in Czech by the first author, while the subsequent analysis and theme development were conducted collaboratively by both authors. Translation into English occurred only after theme development, for the purposes of reporting the findings and presenting illustrative quotations. Both authors contributed to the translation process, drawing on the second author’s extensive experience with Czech–English translation of qualitative data and psychological measures (Zábrodská et al., 2026). Conducting the analysis in the original language was intended to preserve the meanings and nuances of participants’ accounts and minimise interpretive loss associated with translation (Temple & Young, 2004).

### Participants and recruitment

A total of 30 semi-structured interviews were conducted with students enroled in artistic disciplines across Czechia. Participants were recruited from six public faculties and multiple departments and represented three major artistic fields: performing arts (*n* = 13), audiovisual arts (*n* = 9), and visual arts (*n* = 8). The sample included 17 women and 13 men. All participants identified as White, reflecting both the relatively ethnically homogeneous demographic composition of Czechia and the institutional structure of arts higher education, which remains organised predominantly through Czech-language instruction. Most were Czech nationals, alongside five Slovak and two Ukrainian students, consistent with regional migration patterns and linguistic proximity. Most were between 21 and 30 years old, with only two participants aged between 30 and 35. Most had entered higher education shortly after completing secondary school, although some were admitted only after several application attempts. Only one participant was a parent. Detailed information about participant characteristics is summarised in Table I.

Participants were recruited initially through communication channels at participating faculties, including study-related mailing lists, and through social media. Eligibility criteria required current enrolment in a degree programme in the performing, audiovisual, or visual arts. Recruitment messages were shared both in Facebook groups affiliated with individual institutions and in broader groups connecting arts students across institutions. These strategies were later complemented by the authors’ professional and institutional networks and by snowball sampling, through which participants recommended additional potential participants.

**Table I.** Participant characteristics (*N* = 30).^3^

No.	Gender	Ethnicity	Nationality	Field	Degree	Year
1	M	White	Czech	Audiovisual arts	Bachelor’s	3
2	F	White	Czech	Performing arts	Bachelor’s	2
3	F	White	Czech	Audiovisual arts	Bachelor’s	3
4	M	White	Czech	Performing arts	Master’s	1
5	F	White	Czech	Performing arts	Master’s	2
6	F	White	Ukrainian	Performing arts	Master’s	2
7	F	White	Czech	Performing arts	Master’s	2
8	F	White	Czech	Performing arts	Bachelor’s	3
9	M	White	Czech	Performing arts	Bachelor’s	3
10	F	White	Slovak	Performing arts	Bachelor’s	3
11	M	White	Czech	Performing arts	Master’s	2
12	M	White	Czech	Audiovisual arts	Bachelor’s	3
13	F	White	Czech	Audiovisual arts	Bachelor’s	3
14	F	White	Czech	Performing arts	Bachelor’s	3
15	F	White	Czech	Audiovisual arts	Bachelor’s	3
16	M	White	Czech	Performing arts	Master’s	2
17	F	White	Czech	Visual arts	Master’s	2
18	F	White	Slovak	Performing arts	Master’s	2
19	M	White	Czech	Performing arts	Master’s	1
20	F	White	Czech	Audiovisual arts	Bachelor’s	3
21	F	White	Czech	Audiovisual arts	Bachelor’s	2
22	M	White	Czech	Visual arts	Master’s	2
23	M	White	Czech	Visual arts	Master’s	3
24	F	White	Ukrainian	Visual arts	Master’s	3
25	M	White	Slovak	Audiovisual arts	Master’s	1
26	F	White	Slovak	Visual arts	Master’s	1
27	M	White	Slovak	Audiovisual arts	Master’s	2
28	M	White	Czech	Visual arts	Master’s	1
29	F	White	Czech	Visual arts	Bachelor’s	3
30	M	White	Czech	Visual arts	Master’s	4

^3^ The order reflects the interview sequence.

## Ethics, positionality and reflexivity

All participants provided written informed consent prior to participation, including consent for participation, audio-recording, and transcription. The study was conducted in accordance with applicable institutional and national ethical standards, and ethical approval for the study was granted by the Research Ethics Committee, Faculty of Social Sciences Charles University (Mental Health at Art Higher Education Institutions in the Context of Organizational Cultures, no. 91/2023). Participation was voluntary, and participants were informed of their right to withdraw from the study at any time. Interview recordings were transcribed and anonymised and identifying details such as the names of institutions and specific programmes or departments were removed to minimise the risk of participant identification. In the analysis and reporting of the data, participants were identified only through numerical identifiers. All data were treated confidentially and stored securely. All quotations presented in this article were translated from Czech into English and were lightly edited to minimise the risk of identification. These edits included minor adjustments to wording and the removal or generalisation of potentially identifying details (such as distinctive expressions, speech patterns, or specific personal information), while preserving the original meaning of participants’ accounts.

The first author entered the field as an outsider, with no prior background in arts education and no established relationships with art students or staff in the field. However, during the research, she began teaching at one of the arts higher education institutions within the broader field, which shifted her position towards that of an insider. Although this shift provided deeper contextual understanding, it also introduced potential interpretive risks related to familiarity and role expectations. To minimise potential conflicts of interest, no participants were recruited from the institution where the first author taught. The second author, whose expertise lies in occupational health in academia and qualitative methodology (Zábrodská et al., 2018), contributed to the analytic process through ongoing discussion, critical interpretation, and reflexive supervision of the emerging analysis. Reflexive notes and analytic memos were used throughout to document and critically reflect on how the authors’ positions may have influenced data collection and interpretation.

## Data analysis

The data were analysed using reflexive thematic analysis (RTA; Braun & Clarke, 2022) supported by Atlas.ti software. Initial coding and theme development were primarily inductive and grounded in the data. The SPM, Bourdieu’s concepts of capital and field and critical accounts of meritocracy were subsequently used during the later interpretative stages to examine relationships among themes and develop the broader theoretical interpretation. Drawing on Braun and Clarke (2022), the analysis followed their six-phase process, including familiarisation with the data, generation of initial codes across the full dataset, and subsequent development and refinement of themes understood as shared patterns of meaning. Coding focused on meaning units in which participants described situations, interactions, and institutional routines experienced as demanding or burdensome.

Candidate themes were generated through repeated comparisons within and across interviews and were further developed through analytic memo writing and thematic mapping. Throughout the analytic process, the authors moved iteratively between the data, emerging codes, and developing themes to refine interpretations and maintain close engagement with participants’ accounts. The final thematic structure organised the nine themes into three overarching categories of stressors. During the later cross-theme synthesis, the authors examined how the themes related to one another and identified recurring expectations concerning what students were required to tolerate to belong and succeed within the artistic field. The overarching conceptual interpretation presented in the Discussion emerged from this synthesis rather than being used as an a priori analytic category. An illustrative coding matrix showing the progression from initial codes to analytic dimensions, final themes, and overarching categories of stressors is provided in Appendix 1.

Analytic quality and credibility were supported through several strategies, drawing on methodological recommendations for rigorous qualitative enquiry (Cresswell & Báez, 2021): sustained engagement with the data, reflexive journaling and analytic memo writing, iterative comparison between emerging interpretations and the full dataset, attention to contradictory accounts, and ongoing critical discussion between the two authors. These discussions were used to question assumptions, consider alternative interpretations, and deepen the analysis rather than to establish inter-coder agreement, consistent with the principles of RTA (Braun & Clarke, 2021).

## Findings

Using reflexive thematic analysis, we identified three categories of psychosocial stressors: institutional, organisational, and interpersonal. Within each category, three themes were developed, capturing distinct yet interrelated patterns in participants’ experiences of psychosocial stress (see Table II).

**Table II.** Thematic structure of psychosocial stressors: categories, themes, and analytic dimensions.

Categories of stressors	Themes	Analytic dimensions
Institutional stressors	Shared norms of total commitment	• Total artistic devotion • Normalised excessive demands • Commitment under precarity • Cumulative survival burden • Unequal capacity for commitment
	Defining success in the art world	• Meritocratic success ideal • Failure as anticipated norm • Individualised responsibility for outcomes • Narrow hierarchy of artistic legitimacy • Unrecognised alternative career paths
	The social dimension of artistic success	• Networking as an unwritten professional expectation • Unequal access to artistic networks • Networking as imposed self-promotion • Social capital as a protective resource • Maintaining professional visibility
Organisational stressors	The time demands of study	• Time-intensive study regime • Temporal dominance of study • Compressed everyday schedule • Competing time demands • Exhaustion under time pressure
	Evaluation of artistic work	• Ambiguous evaluative standards • Subjectivity of evaluation • Unclear meaning of grades • Emotional feedback • Evaluation and artistic self-doubt
	Experiencing the Final Critique	• Final Critiques as peak pressure • Intensified pre-critique workload • Public exposure through critique • Normalisation of exhaustion during Final Critiques • Unclear Final Critique expectations
Interpersonal stressors	Blurred boundaries in student–teacher relationships	• Familial informality in the study environment • Informality as a source of belonging • Ambiguous relational roles • Underlying power asymmetry • Professional overreach into personal life
	Intergenerational and value-based tensions	• Conflicting artistic values • Institutional dominance of older generations • Resistance to pedagogical change • Lack of recognition for student perspectives • Dismissal of socially engaged topics
	Competitive dynamics among peers	• Competition as pedagogical strategy • Competitive pressure • Rivalry framed as professional preparation • Limited collaborative infrastructures • Prestige hierarchies between departments

### Institutional stressors

#### Shared norms of total commitment

The participants described a pervasive expectation that artistic study should occupy a central place in their lives and that becoming an artist requires total commitment. This expectation was reproduced by teachers, who repeatedly emphasised the necessity of full commitment and framed intensive self-sacrifice as both normal and necessary, as illustrated by the following extracts:

*“When only three people out of a hundred are admitted to a studio, there is an expectation that you will give it everything.”* (P23, Male)

*“Our teacher once told us that they also had to work extremely hard and dedicate themselves fully to it and that we have to be able to manage this as well if we want to study here.”* (P30, Male)

These accounts indicate that total commitment was communicated not merely as a practical requirement of study but as a normative expectation. The selectivity of admission and teachers’ retrospective accounts of their own intensive commitment helped legitimise self-sacrifice as an expected part of artistic training. Beyond the demanding nature of this norm itself, students also described the difficulty of sustaining such expectations under conditions of material and financial constraint. All participants reported working alongside their studies, both for personal and professional development and out of financial necessity, particularly given the high cost of living in urban centres such as Prague and Brno. Under these conditions, the ideal of total artistic devotion was experienced as unevenly attainable. Students from more privileged socioeconomic backgrounds were perceived as having greater freedom to invest time and energy in artistic work, whereas others had to divide their attention between study, paid work, and the practical demands of supporting themselves. As one participant observed,

*“The differences between the students here are very real, even if they are not immediately visible. We all attend the same school, look equally exhausted, and dress similarly. Only later did I realise that a classmate who often borrows money from others for cigarettes actually comes from a very privileged background and can spend the whole exam period abroad. These schoolmates have much more freedom and security. Moreover, many of my other classmates have to work multiple jobs alongside their studies just to make ends meet. And in that situation, it is impossible to fully dedicate yourself to school.”* (P23, Male)

This account further illustrates how the shared norm of total commitment operated as a universal institutional expectation while placing unequal demands on students depending on their material circumstances and access to resources. Socioeconomic background therefore shaped students’ capacity to meet expectations of total devotion, making the same institutional norm substantially more burdensome for some students than for others.

#### Defining success in the art world

The students described repeated messages that promoted a strongly meritocratic understanding of success in the art world, in which individual talent, perseverance, and maximal investment were presented as the key conditions of future recognition. At the same time, this ideal was accompanied by repeated messages that only a very small minority of graduates would actually succeed. One participant described how this narrative structured expectations from the outset of study:

*“Among the students, there is a widespread expectation that only a small minority will succeed, while most will face significant difficulties. This message is communicated early in the study experience and continues to be reinforced through an ongoing rhetoric of scarcity and limited chances of success.”* (P22, Male)

The meritocratic framing of success individualised responsibility for academic and professional trajectories by locating it primarily at the level of individual students, contributing to the internalisation of failure as a personal shortcoming while obscuring the role of structural conditions. As reflected in the students’ accounts, success was narrowly defined in teachers’ narratives, not just as meaningful artistic work but also as visible recognition and the ability to sustain oneself exclusively through artistic production.

Alternative trajectories, such as combining artistic practice with cultural-sector work or teaching, were not recognised as equally legitimate options within the school environment. As one participant noted,

*“I felt that the highest success was to be an exhibiting artist of the classical kind. And that all other activities basically mean that you’re not a ‘real’ artist.”* (P24, Female)

This configuration made success appear both highly valued and highly inaccessible, intensifying uncertainty about professional futures and encouraging students to interpret possible failure in individual rather than structural terms, thereby creating a significant source of stress.

#### The social dimension of artistic success

The participants described receiving repeated messages that success in the arts depends not only on artistic work but also on the ability to build and maintain social connections. Networking was described as an unwritten but highly consequential part of artistic education, an expectation communicated from the beginning of their studies through formal and informal interactions with teachers, peers, and the broader artistic community. Networking therefore operated as an unwritten professional expectation through which students learned that visibility, personal contact, and social participation were crucial for future opportunities.

However, access to such networks was distributed unequally. Students without a prior artistic background had to develop professional networks gradually, a process they described as time-consuming, uncomfortable, and psychologically demanding. These students reported persistent anxiety about forming relationships, uncertainty about potential collaborations, and fear of exclusion from informal networks through which important projects, commissions, and opportunities circulated. Some students experienced the need for networking as externally imposed and in conflict with their understanding of artistic practice. One participant explained this tension as follows:

*“I don’t feel comfortable with it. I’m an introvert; I want to devote myself to my field, but seeking out and building new connections doesn’t feel natural to me. It feels like selling myself, which makes me uncomfortable.”* (P28, Male)

In contrast, students from families already embedded in the artistic field—for example, those whose parents worked as artists, or in other cultural professions—often entered arts higher education with pre-existing contacts, including with teachers, and greater familiarity with how the field operated. Their family background thus provided an important source of social capital, giving them access to networks, tacit knowledge, and professional relationships that other students had to build gradually during their studies. For these students, social relations were a more natural part of their study and professional trajectories, facilitating collaboration, strengthening a sense of belonging, and increasing confidence in future prospects. Students without such family connections, by contrast, had to acquire this social capital themselves, making networking an additional demand rather than a resource they could draw on from the outset.

The data also illustrated how care responsibilities could restrict participation in the informal social settings through which professional relationships and opportunities were developed. This was particularly visible in the account of the participant who had returned to study after maternity leave:

*“In a way, this is part of the profession; contacts and projects are discussed informally in social settings. And once you’re not part of this social life, you’re out. I felt this very strongly when I was coming back after maternity leave. I deliberately went to events I didn’t even want to attend because I knew many people would be there and I needed to be seen.”* (P7, Female)

In participants’ accounts, social capital thus functioned both as a resource and as a source of inequality, with important consequences for students’ confidence and perceptions of their future prospects in the artistic field. Networking operated not simply as an additional career demand but as an implicit expectation concerning the conditions of artistic success: students learned that talent and artistic work alone were insufficient without visibility, relational investment, and access to professional networks.

### Organisational stressors

#### The time demands of study

The participants described their studies as highly time-intensive and difficult to combine with other important aspects of life, such as paid work and personal life. Especially in earlier years of study, lectures often extended across most weekdays, from morning to evening, and were supplemented by workshops, block teaching, or special events, often extending into weekends. Students typically described their studies as the dominant organising principle of everyday life, as illustrated in the following extracts:

*“Right now, my week looks like this: on Mondays I finish at school at six, then I go to tutoring, and I get home around eleven. On Tuesdays I have school until six, then I have a two-hour break where I do my own work or tutor. … It’s all very tight. Fitting everything in.”* (P2, Female)

*“We usually have school until six. On some days, I work evenings in a bar because that job makes it possible to combine things. Sometimes I finish work early in the morning and then go back to school again.”* (P19, Male)

The study burden was intensified by the fact that most students also needed to engage in paid work alongside their studies, often out of financial necessity, as discussed above. Flexible jobs in hospitality or the cultural sector made this combination possible, but often at the cost of sleep, recovery, and longer-term well-being. The students linked this regime to chronic fatigue, a reduced sense of autonomy over their time, reduced creativity in their artistic work, and strain in personal relationships.

The temporal organisation of study thus emerged as more than a problem of excessive workload: it constated a pervasive condition that organised students’ everyday lives around competing demands, limited opportunities for recovery, and ongoing exhaustion. This time-intensive regime was also associated in some participants’ accounts with maladaptive coping strategies, including alcohol or other substance use, which they described as ways of managing chronic psychological and physical demands, as illustrated in the following extract:

*“Drugs, drugs helped me a lot in coping with [the demands of studies]. Alcohol in the earlier years…, and then drugs simply became part of my life - cocaine, MDMA, for instance. Although cocaine isn’t exactly a stress-relief drug, it gives you a certain sense of confidence. That’s how it worked for me. You do a line and suddenly you feel like everything is fine.”* (P16, Male)

#### Evaluation of artistic work

The participants described the evaluation of artistic work as one of the most stressful aspects of their studies. They often experienced feedback from teachers as ambiguous or even contradictory, highly subjective, and difficult to translate into meaningful guidance. This experience became particularly salient in situations in which institutions required formal grading of student performance while framing artistic evaluation as inherently subjective, a claim frequently emphasised by teachers. The following extract illustrates how the absence of clearly defined criteria made it difficult for students to interpret their evaluations meaningfully:

*“The teachers keep telling us that our field is subjective, that it is judged subjectively and cannot be evaluated objectively. But then we get grades. Someone gets an A, someone a B, someone a C. And then we ask why. I’m fine with getting a C, but does it mean I need to improve? So I ask and suddenly no one can really tell me.”* (P5, Female)

The stressor therefore appeared to lie not in the inherent subjectivity of artistic judgement itself, but in its combination with unclear criteria, limited explanatory feedback, and students’ dependence on individual teachers’ evaluations. The students also described emotionally charged forms of feedback that they experienced as hurtful, humiliating, or devaluating. In such situations, evaluation was perceived as a judgement not only of the artistic work but of the self more broadly. This perception was especially pronounced in performing arts disciplines, in which the assessed performance is often inseparable from the students’ bodies, voices, or modes of expression. One participant described feedback that crossed into overt disparagement:

*“A student performs and is supposed to get feedback from the teacher. But the teacher may just sit there and spend ten minutes sarcastically saying, ‘Oh, you’re really an actress…’. Or they just tell you that you are completely stupid and what you are doing is just crap. This is what their feedback can look like.”* (P8, Female)

These accounts suggest that ambiguous and highly subjective assessment practices generated considerable uncertainty about how students should understand the quality of their work and their own artistic competence. Students also experienced such practices as reinforcing power asymmetries in student–teacher relations and undermining their confidence, sense of belonging, and artistic identity. This negative impact appeared particularly pronounced in the early stages of their studies, especially when bodily expression was involved. While still finding evaluation emotionally challenging, more senior students appeared increasingly to accept such uncertainty as integral to performing arts training, suggesting one possible route through which evaluative ambiguity could become normalised over the course of study.

#### Experiencing the Final Critique

Final Critiques are end-of-semester evaluations during which students present work created over the course of the semester. Traditionally, Final Critiques take place in front of teachers and peers, but they are often open to a wider public, requiring both professional and personal self-presentation. These evaluations were described as particularly intense and stressful aspects of arts higher education. The participants portrayed them as periods of peak pressure in which evaluation, public presentation, and extreme workload converged. Preparation often involved long hours of work, sleep deprivation, and the compression of multiple academic demands at the end of the semester. One participant described this period as follows:

*“During the last of the Final Critiques, I only managed to go home to shower. I had four Final Critiques in a row, and every time I worked at school overnight. The week before the Final Critiques, we stayed at school until about ten in the evening. Then, we went home, to get some sleep at least.”* (P3, Female)

The students also emphasised the personal nature of the work presented during the Critiques. Because their artistic work often drew on their personal experiences, emotions, and values, public evaluation could be experienced as exposing the students themselves as well as their work. This pressure was further intensified by unclear and shifting expectations. The students described the procedures and criteria governing Final Critiques as often insufficiently specified, forcing them to learn the rules through trial and error. Their timing at the end of the semester further intensified the pressure, as students were simultaneously required to complete other academic obligations and examinations. The following quotation illustrates the extreme work intensity and its physical toll:

*“Many of us sleep at school and work overnight before Final Critiques because we can’t keep up. (…) The teachers enjoy walking through the studios in the evening and watching how we work. If you don’t sleep one night, you collapse the next day.”* (P5, Female)

This account also suggests that the students’ exhaustion was not only tolerated but could become actively normalised within the educational environment. The presence of teachers in the studios during late hours, together with the apparent acceptance of such work intensity, may reinforce the idea that working at the limits of one's physical and mental capacity is an expected and legitimate aspect of artistic education. In some programmes, Final Critiques were also described as carrying the risk of study termination. Participants reported that failure to pass a Final Critique could result in dismissal from the programme, further amplifying the pressure and uncertainty that accumulated during this period. The Final Critiques thus emerged as a concentrated expression of broader features of arts training: extreme workload, public exposure, unclear evaluative standards, the normalisation of exhaustion, and dependence on subjective teacher evaluation.

### Interpersonal stressors

#### Blurred boundaries in student–teacher relationships

The participants often described the study environment as informal, intimate, and, at times, familial. Small cohorts within particular programmes, year groups, or studios, combined with sustained daily contact, fostered close relationships between students and teachers, which many participants valued as a source of community, belonging, and openness. Students commonly reported joint activities with teachers outside the formal educational setting, such as social gatherings in pubs, informal trips, or collaboration on projects outside the institution. This familial atmosphere was widely perceived as a positive aspect of study, contributing to a sense of security and a home-like climate within the institution.

However, informality also created uncertainty about relational boundaries. Although relationships could feel friendly and personal, students remained institutionally subordinate, making the boundaries of appropriate student–teacher interaction difficult to determine. Some students described situations in which informal relationships with teachers generated uncertainty about potentially negative consequences when relationships became strained, disagreements emerged, or interpersonal dynamics changed, as illustrated in the following quotation:

*“These teachers definitely create the environment on the edge. So it’s necessary for students to be aware of this and understand that it can have negative consequences if the relationship becomes very friendly.”* (P17, Female)

These accounts highlight how informality could obscure rather than eliminate the underlying power asymmetry, making it more difficult for students to recognise and navigate relational boundaries. This ambivalence was intensified by the fact that teachers often occupied important positions within the artistic field − for example, as directors, curators or actors − and could therefore significantly influence students’ future opportunities. The teachers’ professional standing and authority extended beyond the educational setting, further increasing students’ dependence on these relationships. One participant described how this blurring of educational and personal boundaries could become particularly intrusive:

*“A teacher once told me informally that they had been reluctant to accept me and had serious doubts about my presence in the department, fearing that I might negatively affect the collective. At another point, the same person commented on my intimate relationship with another student, questioning whether I was behaving appropriately and giving my partner enough space. After the relationship ended, the teacher repeatedly crossed professional boundaries.”* (P10, Female)

Such accounts illustrate how relational closeness could coexist with, and potentially conceal, persistent institutional hierarchy, placing students in an ambiguous and shifting terrain of intimacy and dependence that could increase their vulnerability when relational boundaries were crossed.

#### Intergenerational and value-based tensions

Intergenerational differences in understandings of higher education and artistic work emerged as a prominent theme in the interviews. The participants described recurrent tensions between students and older generations of teachers, particularly around artistic values, definitions of success, pedagogical approaches and expectations, and the social role of art. Although younger generations of teachers have gradually entered arts higher education institutions, participants perceived senior and leadership positions as still being predominantly occupied by older academics with long institutional tenure, giving them substantial influence over institutional and pedagogical decisions. Older faculty members were often perceived as holding considerable institutional authority while being resistant to new artistic approaches, teaching methods, or socially engaged topics. As one participant observed,

*“There are always the older professors in leadership positions. Or even if they are not in the leadership position, they are the oldest faculty with a vision of theatre that is different. And they are unable to accept new methods or teaching plans.”* (P16, Male)

These accounts suggest that generational differences became particularly consequential when differences in artistic or pedagogical values coincided with asymmetries in institutional authority. Students could therefore experience disagreement not simply as a difference of opinion, but as a situation in which their emerging artistic perspectives received limited recognition from those with considerable influence over their education. These intergenerational tensions became particularly salient when students sought to address contemporary social issues in their artistic work, such as gender identity and transition, women in arts leadership positions, or inequalities in cultural institutions. Students perceived these topics as important and regarded their inclusion into artistic practice as meaningful. However, participants described such themes as frequently being dismissed as irrelevant or misunderstood by teachers, contributing to feelings of not being understood or recognised and to distrust in the evaluative environment. One student recalled presenting a bachelor’s thesis topic on women in leadership and being told that the issue was “completely irrelevant”:

*“When I first presented my topic for my bachelor’s thesis, the teacher’s classic response was, ‘But there are plenty of women in leadership; it’s completely irrelevant,’ and then they started listing specific names. But we couldn’t understand each other in terms of what my argument was and why I found the topic important. And we didn’t understand each other even when I submitted my work to some other teachers. And, as usual, these were the older teachers.”* (P15, Female)

For students, such tensions had implications beyond disagreements over content. Because their artistic work was often closely tied to personal values and emerging professional identities, a lack of recognition in these areas could undermine their sense of legitimacy, motivation, and belonging within the institution. Some participants responded by disengaging from teaching situations they experienced as dismissive or hostile. In relation to a teacher who repeatedly introduced provocative views on gender and generational issues into class discussions, one student described the following:

*“He kept trying to provoke discussion by throwing out controversial opinions. Much of the time, he was talking more about his own worldview than about what we were supposed to discuss in class. It was just unpleasant to sit there and listen. We all agreed that we basically just went to sit through the class, get the credit, and forget about it.”* (P12, Male)

Such accounts suggest that intergenerational and value-based tensions could lead to students’ withdrawal, emotional distancing, and reduced engagement with study, especially when differences in perspective were embedded in relationships characterised by unequal institutional authority.

#### Competitive dynamics among peers

The prestige and scarcity narratives described above also structured peer relations. Admission to highly selective arts programmes initially generated a strong sense of achievement, distinctiveness, and motivation, but this was accompanied by repeated messages that only a small proportion of graduates would ultimately succeed in the art world. Combined with a strong emphasis on individual performance, these narratives transformed professional uncertainty into a source of competitive pressure among students. Some participants described peer rivalry as actively reinforced by teachers and framed as preparation for entry into the art market, as illustrated by the following extract:

*“It felt like the teacher deliberately created a competitive environment to prepare students’ work and the students themselves for entering the art market. Which didn’t suit me at all. (…) It was entirely dependent on the teacher’s approach, giving students the sense that they are unique personalities who must develop their own, in quotes, ‘genius’.”* (P18, Female)

Competition therefore emerged not only spontaneously from peer relations but could also be actively produced and legitimised through pedagogical practices as preparation for professional artistic life. In visual and audiovisual arts, participants described the study process as highly individualised. Work was typically organised around independent projects, with limited institutional support for collaboration. This situation intensified students’ feelings of isolation and indirect rivalry, as they constantly compared their work, pace, and achievements with those of their peers. As one participant commented,

*“Everyone is doing their own thing, working individually on their own projects. We don’t really have any group work, and it isn’t supported in any way. So it can really feel as if each of us is on our own, because there is no real space for collaboration, even though I think that would have been nice.”* (P22, Male)

In contrast, performing arts disciplines, such as acting or directing, relied more heavily on cooperation, yet competition was not absent. Participants described substantial variation across disciplines and programmes, with informal hierarchies shaped by institutional history, prestige, and the authority of particular faculty members. These hierarchies created uneven conditions and reinforced competitive dynamics not only between individual students but also between programmes and artistic fields.

## Discussion and conclusion

This study examined psychosocial stressors embedded in the institutional, organisational, and interpersonal conditions of Czech arts higher education. The findings showed that students’ stress could not be understood solely as a response to individual study demands or isolated stressful events; rather, it was consistently embedded in prevailing institutional norms, pedagogical relationships, and unequal conditions of recognition, as also indicated by other studies in arts higher education (Matei & Ungureanu, 2026). Beyond identifying the three interrelated categories of psychosocial stressors, the main contribution of the study lies in uncovering a broader process through which recurring exposure to these stress-inducing conditions communicated expectations about legitimate artistic conduct. Our analysis led us to conceptualise this process as a “hidden curriculum of endurance”: an informal process of professional socialisation through which students learn that tolerating excessive demands, uncertainty, self-sacrifice, evaluative ambiguity, professional insecurity, and relational dependence is both normal and, to some extent, necessary for becoming and being recognised as a legitimate participant in the artistic field. We use “endurance” to refer to this broader learned expectation of tolerating and persisting through psychosocial demands as part of legitimate artistic formation, extending well beyond persistence under high workload. The “hidden curriculum of endurance” represents the process through which repeated encounters with these stressors conveyed implicit lessons about what becoming an artist requires and contributed to their gradual normalisation as expected features of artistic training and professional socialisation.

This interpretation emerged from the analysis rather than serving as an a priori theoretical framework and led us to revisit the established concept of the “hidden curriculum” in the sociology of education (e.g., Hafferty & Castellani, 2009). The concept refers to norms, values, and expectations communicated largely outside the formal curriculum through pedagogical practices, assessment, interpersonal relationships, institutional routines, and professional norms (Koutsouris et al., 2021; Lempp & Seale, 2004; Rossouw & Frick, 2023; Sambell & McDowell, 1998). Here, “hidden” does not imply that these expectations are deliberately concealed or necessarily unconscious. Rather, “hidden” refers to taken-for-granted and insufficiently articulated assumptions embedded in everyday educational practices, through which students infer what institutions actually value and what kinds of conduct and professional identities are expected of them (Hafferty & Castellani, 2009; Koutsouris et al., 2021). As shown in our study, some elements of the hidden curriculum were communicated quite explicitly (for example, when teachers recounted having themselves endured extreme workloads), while the broader lesson that such endurance represented an appropriate or necessary part of artistic formation remained largely implicit.

The findings identified several interconnected mechanisms through which this hidden curriculum was transmitted. First, teacher modelling and retrospective narratives legitimised intensive work and self-sacrifice by presenting them as established features of artistic training. Second, assessment practices − particularly ambiguous and highly subjective evaluation and Final Critiques − exposed students to evaluative uncertainty, public scrutiny, and concentrated pedagogical authority. While some degree of subjectivity may be inherent to artistic assessment, unclear criteria and limited explanatory feedback can make critique difficult to interpret and heighten students’ dependence on evaluators (Blair, 2007; Cleary Rodrigues, 2025; Motley, 2017). In line with Sambell & McDowell, (1998) argument that assessment communicates messages beyond its formal evaluative function, these practices taught students not only what counted as legitimate artistic quality but also how they were expected to respond to uncertainty, critique, and emotional exposure. Third, institutional narratives of selectivity, prestige, and scarcity legitimised total and potentially excessive commitment. Finally, informal student–teacher relationships, networking expectations, and competitive peer cultures communicated that artistic merit alone was insufficient: successful participation also required navigating relational dependencies, maintaining visibility, and continually investing in professional networks.

These mechanisms may explain how exposure to particular stressors could lead to their gradual normalisation. Repeated encounters with excessive demands, opaque evaluation, professional uncertainty, competition, and dependence on informal relationships communicated expectations about legitimate artistic conduct. Such recurring exposure could make these demands appear characteristic of the artistic field, while the capacity to tolerate them could itself signify dedication and suitability for an artistic career. Practices such as sleep deprivation before Final Critiques, chronic overcommitment, or continual investment in professional visibility could therefore become understood as ordinary or even constitutive features of becoming an artist. Similar patterns of intensive and discipline-specific workloads and stress have been also documented in higher music education (Jääskeläinen et al., 2023; Jääskeläinen, 2022). Importantly, however, the hidden curriculum research emphasises that students actively interpret rather than simply absorb implicit messages (Sambell & McDowell, 1998). Accordingly, this process was neither uniform nor deterministic: some participants in our study questioned excessive demands, resisted networking expectations, or disengaged from relationships they perceived as harmful. The hidden curriculum of endurance should therefore be understood as a normative process of professional socialisation rather than as a uniform outcome.

The expression of these pressures also varied across artistic disciplines and programme structures. Performing and audiovisual arts were characterised by more highly structured schedules and time-intensive collaborative activities, whereas visual arts generally offered greater autonomy while placing more responsibility for artistic productivity and time management on individual students, as also observed in research on student autonomy and self-direction in studio-based visual arts education (Edström, 2008). Social experiences appeared to depend less on discipline itself than on programme organisation: collaborative programmes tended to mitigate competition through teamwork, while programmes centred on individual artistic production were associated with greater isolation, individual responsibility, and more direct peer competition. Despite these differences, expectations of total commitment, networking, and the emotional toll of artistic critiques were shared across disciplines. This combination of disciplinary variation and shared normative expectations is consistent with research into aesthetic socialisation as a process through which students learn the aesthetic, occupational, and identity-related norms of becoming an artist (Fang, 2020), as well as with recent evidence that institutional cultures in higher music education shape students’ emerging professional identities alongside their well-being (Matei & Ungureanu, 2026). Our findings extend this perspective by identifying endurance itself as an additional normative dimension of artistic formation.

The hidden curriculum identified in our study did not represent a fully coherent set of values but contained several important tensions. The first concerned the coexistence of meritocratic and relational constructions of artistic success. Students were socialised into a meritocratic ethos in which success was presented as the outcome of individual talent, discipline, effort, and total commitment to artistic work. At the same time, they also learned that success depended on their capacity to build professional networks, maintain visibility, and adapt to the field’s unwritten rules, a dynamic also highlighted by Goodwin and Vincent (2024) and Skála and Chylíková (2022). In this sense, artistic success emerged as dependent on the accumulation and mobilisation of social capital, as suggested by Bourdieu and critical accounts of meritocracy (Bourdieu, 1993, 1986; Brook et al., 2020; Littler, 2017). The hidden curriculum consequently had unequal implications. Students from artistic families or with greater economic security entered their studies with resources, networks, and tacit knowledge that facilitated compliance with expectations of intensive commitment and professional visibility. By contrast, students who had to combine intensive study with paid employment or lacked pre-existing artistic networks had to invest additional time and effort in acquiring these resources. Expectations presented as universal markers of artistic seriousness were therefore substantially easier for some students to meet than for others, paralleling broader accounts of the hidden assumptions surrounding the “ideal” higher education student (Koutsouris et al., 2021).

Another contradiction of the hidden curriculum concerned the coexistence of prestige and precarity. Admission to a highly selective arts programme conferred profound symbolic value and helped to legitimise expectations of exceptional commitment, while students were simultaneously reminded that, notwithstanding their effort, only a minority of graduates would sustain themselves solely through artistic work. Anticipated professional insecurity therefore appeared to reinforce rather than challenge the hidden curriculum: scarcity provided a rationale for learning early to tolerate intensive work, competition, and uncertain recognition. This dynamic echoes broader analyses of cultural labour, in which symbolically attractive and meaningful work is frequently intertwined with precarity, individualised responsibility, and the normalisation of insecurity (Banks, 2017; Gill & Pratt, 2008; Jaramillo-Vázquez, 2026). The correspondence with international research suggests that this mechanism may extend beyond Czechia, although its specific manifestations are likely to remain dependent on national and institutional context.

The concept of a hidden curriculum of endurance also brings the study’s theoretical perspectives into a coherent framework. The SPM highlights that exposure to stressors is socially organised and unevenly distributed rather than merely individual or situational (Pearlin et al., 1981). In this study, institutional, organisational, and interpersonal stressors represented interrelated pressures embedded in a study environment marked by unequal access to resources and recognition. Bourdieu’s framework further helps explain how unequal economic, social, and cultural resources shape students’ capacity to navigate these expectations and convert participation into recognition (Bourdieu, 1993, 1986; Heffernan, 2022; Naidoo, 2004). Critical accounts of meritocracy illuminate how such inequalities can become obscured when artistic success is represented primarily as the product of individual talent and commitment (Brighouse, 2022; Littler, 2017; Liu, 2011). The hidden curriculum adds a processual dimension: it explains how these socially structured and unequally attainable expectations are communicated through everyday educational practices and gradually normalised as requirements of legitimate artistic conduct. In this sense, the study extends an SPM-informed understanding of psychosocial stress by identifying professional socialisation as one mechanism through which socially patterned stressors may become culturally legitimised and incorporated into emerging professional identities.

The findings have important implications for policy and practice. Responses to student mental health in arts higher education should extend beyond individualised support to address the pedagogical and organisational practices through which psychological stress may be produced and normalised. More transparent assessment practices, clearer relational boundaries, and institutional support that acknowledges unequal conditions of artistic training appear particularly important. Institutions could also make implicit expectations concerning workload, networking, artistic success, and evaluation more explicit and open to critical discussion. Challenging the assumption that exhaustion, constant availability, or tolerance of ambiguity constitute evidence of artistic seriousness may help foster more sustainable forms of professional education in which becoming an artist does not require students to normalise conditions detrimental to their well-being.

## Limitations and future directions

This study has several limitations. First, the context-specific nature of this qualitative study should be considered when interpreting its findings. The study provides an in-depth account of psychosocial stress within the relatively small, selective, and interconnected field of Czech arts higher education. Although parallels with international studies discussed above suggest that some of the processes identified here may extend beyond Czechia, their expression may vary across institutional, disciplinary, and national contexts. Future cross-national research could therefore examine the transferability of the hidden curriculum of endurance. Second, self-selection in recruitment may have resulted in greater participation by students for whom psychosocial stress or problematic study experiences were particularly salient. The study therefore cannot establish the prevalence of the identified experiences. Moreover, although the sample included students from visual, audiovisual, and performing arts and allowed us to identify some disciplinary variation, the number of participants within individual fields did not permit systematic discipline-specific comparisons. Larger qualitative studies could examine disciplinary variation in greater depth, while quantitative or mixed-method research could assess the prevalence and distribution of the identified stressors across different forms of arts training. Finally, the study examined students’ accounts at a particular point in time and did not include the perspectives of teachers or other institutional actors. Because the data were cross-sectional, the proposed process of normalisation should be understood as an interpretative account rather than as direct observation of change over time. Multi-perspective research could clarify how expectations embedded in the hidden curriculum are communicated and negotiated within arts higher education. Longitudinal qualitative research would be particularly valuable for examining the process conceptualised here: whether and how repeated exposure to these expectations contributes to their gradual normalisation and incorporation into emerging professional identities.

## Data Availability

The data that support the findings of this study are not publicly available due to ethical and confidentiality considerations. The dataset consists of qualitative interview material, and even after anonymization, there remains a risk of participant identification given the relatively small and interconnected institutional context. Data sharing is therefore restricted in order to protect participant confidentiality and to comply with the conditions of informed consent.

## Endnotes

^1^
For example, 66 out of 486 applicants were admitted to the Film and TV School of the Academy of Performing Arts in Prague (14%), while the Theatre Faculty of the Janáček Academy of Performing Arts admitted 78 out of 335 applicants (23%). The Academy of Fine Arts in Prague accepted 42 out of 305 applicants (14%), and the University of Applied Arts in Prague admitted 65 out of 684 applicants (10%) (VysokeSkoly.com, 2025).

^2^
Czechia’s spending on educational institutions (primary to tertiary) has consistently remained below the OECD average, at 4.6% of GDP in 2023 compared with the OECD average of 4.9% (Organisation for Economic Co-operation & Development, 2024).

## Generative artificial intelligence (AI)

Artificial intelligence (AI) tools were used solely to support language editing, stylistic refinement, and literature organisation during manuscript preparation. Specifically, ChatGPT (OpenAI, GPT 5.5) was used for grammar and stylistic suggestions, while NotebookLM (Google, latest available version) assisted in organising literature sources. No AI tools were used for data analysis, interpretation of results, or generation of scientific conclusions. The authors take full responsibility for the content of the manuscript.

## Appendix 1. Illustrative coding matrix

The matrix illustrates the analytic progression from initial codes to broader analytic dimensions and final themes, which were iteratively compared and organised within three overarching categories of psychosocial stressors. A subsequent cross-theme synthesis informed the overarching conceptual interpretation of the hidden curriculum of endurance.

**Table:**

Initial codes → Analytic dimensions	→ Final themes →	Stressor categories → Hidden curriculum of endurance
INSTITUTIONAL STRESSORS		
Initial codes (examples)	Analytic dimensions	Final theme
• Expectation of devotion • Study–work–life balance • Normalisation of overwork • Art as a commitment • Prioritisation of studies • Studying beyond personal limits • “You have to sacrifice yourself for art”	• Total artistic devotion • Normalised excessive demands • Commitment under precarity • Cumulative survival burden • Unequal capacity for commitment	**Shared norms of total commitment**
• Perceptions of success and failure in art • Precarious work • Low chances of success • Low career expectations • The narrative that artists are bound to struggle • Definition of success	• Meritocratic success ideal • Failure as anticipated norm • Individualised responsibility for outcomes • Narrow hierarchy of artistic legitimacy • Unrecognised alternative career paths	**Defining success in the art world**
• Social connections • Networking • Self-promotion • Informal networking • Visibility in the art world • Unequal opportunities • “You make connections at the pub”	• Networking as an unwritten professional expectation • Unequal access to artistic networks • Networking as imposed self-promotion • Social capital as a protective resource • Maintaining professional visibility	**The social dimension of artistic success**
ORGANISATIONAL STRESSORS		
**Initial codes (examples)**	Analytic dimensions	Final theme
• Demands of studying during the semester • Class schedule • No personal life • Demanding routines • Insufficient rest • Overload • Chronic fatigue	• Time-intensive study regime • Temporal dominance of study • Compressed everyday schedule • Competing time demands • Exhaustion under time pressure	**The time demands of study**
• Unclear assessment criteria • Subjectivity in assessment • Vulgar feedback • Sense of injustice • Damage to relationships • Lack of time on the part of teaching staff • Feeling devalued	• Ambiguous evaluative standards • Subjectivity of evaluation • Unclear meaning of grades • Emotional feedback • Evaluation and artistic self-doubt	**Evaluation of artistic work**
• Most demanding period of study • “Before final critiques, you basically don’t sleep” • Normalisation of overwork • Pushing performance to the limit • Insufficient feedback • Intense workload • Need for self-promotion	• Final Critiques as peak pressure • Intensified pre-critique workload • Public exposure through critique • Normalisation of exhaustion during Final Critiques • Unclear Final Critique expectations	**Experiencing the Final Critique**
INTERPERSONAL STRESSORS		
**Initial codes (examples)**	Analytic dimensions	Final theme
• Informal meetings with teachers • “We were like a family” • Teachers socialising with students • Inappropriate behaviour • Admiration of teachers • Disappointment with teachers • Unclear professional boundaries	• Familial informality in the study environment • Informality as a source of belonging • Ambiguous relational roles • Underlying power asymmetry • Professional overreach into personal life	**Blurred boundaries in student–teacher relationships**
• Conflicts over the direction of arts education • Intergenerational conflict • Gender imbalance among teaching staff • Devaluation of students’ opinions • Distrust of teachers’ conduct • Dismissal of students’ needs	• Conflicting artistic values • Institutional dominance of older generations • Resistance to pedagogical change • Lack of recognition for student perspectives • Dismissal of socially engaged topics	**Intergenerational and value-based tensions**
• Lack of teamwork • Isolation • Promotion of individualism • Competitiveness • Creation of a hostile environment • Inequalities between departments • Competitive environment	• Competition as pedagogical strategy • Competitive pressure • Rivalry framed as professional preparation • Limited collaborative infrastructures • Prestige hierarchies between departments	**Competitive dynamics among peers**

Note: The initial codes shown are illustrative examples selected to demonstrate the analytic progression and do not represent the full codebook.
